# Healthcare professionals’ knowledge, attitude and acceptance of influenza vaccination in Saudi Arabia: a multicenter cross-sectional study

**DOI:** 10.1186/s12913-019-4054-9

**Published:** 2019-04-15

**Authors:** Thamir M. Alshammari, Kazeem B. Yusuff, Muhammad Majid Aziz, Gehad M. Subaie

**Affiliations:** 1 College of Pharmacy, University of Hail, P.O. Box 6166, Hail City, zip code 81442 Saudi Arabia; 2 Saudi Food and Drug Authority, Riyadh, Saudi Arabia; 3 Medication Safety Research Chair, King Saud University, Riyadh, Saudi Arabia; 4 College of Clinical Pharmacy, King Faisal University, Al-ahsa, Saudi Arabia; 5 School of Pharmacy, Xi’an Jiaotong University, Xi’an, China

**Keywords:** Healthcare professional, Influenza vaccination, Knowledge, Attitude, Saudi Arabia

## Abstract

**Background:**

All healthcare professionals (HCPs) are at high risk of influenza infection. Therefore, immunization is recommended for all HCPs. Due to safety and effectiveness concerns, HCPs have a low vaccination rate. This study was designed to explore the attitude, awareness and knowledge of HCPs toward vaccination for influenza.

**Method:**

A cross-sectional study was performed during October–November 2016. A total of 405 questionnaires were distributed in 8 major hospitals in Saudi Arabia. A validated questionnaire consisting of 31 questions and 5 sections was administered. Statistical Analysis Software (SAS®) version 9.2 was used to analyze the data.

**Results:**

A total of 364 HCPs responded to the study survey, which is a response rate of 90%. A large proportion (61.8%) of participants were female. The majority of the participants were nurses (60.4%). More than half of the respondents (57.7%) were working in government-run hospitals. Among all the participants, approximately 67.6% of HCPs were vaccinated. The majority (84.1%) of HCPs believed that influenza vaccine prevents the flu. Furthermore, approximately 75% of participants believed that HCPs can be more susceptible to influenza infections than other people. The majority of participants (89.6%) knew the proper signs and symptoms of influenza. HCPs’ belief that vaccination prevents influenza infection (OR = 3.93, 95% CI = 1.97–7.82), their awareness of the Scientific Committee for Influenza and Pneumococcal Vaccination (SCIPV)‘s guidelines (OR = 2. 13, 95% CI = 1.16–3.90) and the presence of the standing orders regarding influenza vaccine (OR = 1.57, 95% CI = 1.01–3.21), were the predictors for receipt of influenza vaccine by HCPs.

Many (58.0%) respondents believed that vaccine safety concerns is a major barrier to the vaccination of HCPs. Some misconceptions, such as influenza infection due to vaccination (42.3%) and incorrect perceptions about the symptoms of influenza in adults (50.5%), were found.

**Conclusion:**

The acceptance of and participation in influenza vaccination by HCPs in Saudi Arabia appears to have markedly increased in the 2016 season. Continuing evaluation of vaccination practices is necessary, and more training programs are needed in the future.

**Electronic supplementary material:**

The online version of this article (10.1186/s12913-019-4054-9) contains supplementary material, which is available to authorized users.

## Background

Influenza is a pervasive infectious disease that remains a leading public health challenge worldwide [1, 2]. It is estimated that 291,243–645,832 annual deaths are caused by influenza globally [3]. It has become an increasingly important cause of morbidity and mortality, especially for vulnerable groups, such as pregnant women, the elderly and children, and people living with chronic disorders [4, 5]. Influenza is also associated with a significant economic burden attributable to the considerable direct and indirect healthcare costs at the individual, institutional and societal levels [4–6].

Thankfully, potent and safe vaccines are available to combat the seasonal burden associated with influenza infections, and their administration is centered on evidence-based recommendations that ensure the effective and safe use of influenza vaccines [7–9]. Studies have indicated that annual influenza vaccination reduces the mortality and morbidity of healthcare professionals (HCPs) and patients [10, 11]. However, regular adherence to the yearly schedule of influenza vaccination, widespread acceptance and regular uptake of influenza vaccines and good vaccination coverage have proved challenging, and a variety of factors underlie these phenomena [12, 13]. These factors include hesitancy and aversion to vaccines, concerns about vaccine safety, misinformation/unfounded rumors, poor perception and/or inadequate knowledge of the direct and indirect health benefits associated with vaccinations and poorly informed vaccine-related decision-making by patients and HCPs [14–16]. HCPs play an important role in immunization through their appropriate knowledge, positive attitude, and ample information [17].

A major factor that is consistently associated with patients’ acceptance and uptake of vaccinations, compliance with vaccination schedules and reduction of hesitancy/aversion is the attitude and use of vaccinations by HCPs [17, 18]. In addition, vaccinated HCPs also have a noticeable effect on patients’ decision to immunize [19].

It is well reported that HCPs who have an unfavorable attitude, aversion or hesitancy to vaccinations pass off these vaccination-unfriendly attitudes to patients and tend to recommend vaccinations less frequently [20, 21]. Furthermore, the level of vaccine hesitancy among HCPs has been consistently linked to hesitancy and aversion to vaccination among patients and/or the general population [17, 22]. In addition, the quality, content and delivery of vaccine-related counseling/educational information by HCPs has proven valuable in enhancing patients’ acceptance of vaccinations, reducing hesitancy, boosting vaccination coverage and guiding informed vaccination-related decisions [11, 16]. This is because patients often trust and rely on HCPs for information related to vaccines and vaccine-preventable diseases, and the therapeutic and public health benefits associated with vaccination [16, 23]. It is therefore important to conduct regular appraisal of HCP’s attitude toward, acceptance of and participation in immunization, as these have been established as accurate predictors of acceptance and vaccination rate among patients and/or the general population [24]. This is particularly important for highly infectious seasonal vaccine-preventable diseases, such as influenza, especially in a setting such as Saudi Arabia, which hosts the yearly Hajj, which is the world’s largest gathering, with approximately three million people [25]. This is arguably the only event with the highest density of people from different parts of the globe in such close proximity, a factor that increases the risk of influenza transmission ab initio [26]. Furthermore, Saudi Arabia carries a significant burden of chronic noncommunicable diseases, such as diabetes, hypertension and other cardiovascular diseases, and these are patient groups that have increased susceptibility to the transmission of influenza infection [4, 9, 27]. In addition, the frequent interactions of HCPs with patients and the close proximity within which they deliver healthcare services, especially in an institutionalized care setting, make both parties highly susceptible to the transmission of an infectious disease such as influenza [7, 8]. Hence, the inherent peculiar local susceptibility factors in Saudi Arabia, which increase the risk of influenza infections and the critical roles played by HCPs in enhancing vaccinations by patients, warrant regular assessment of the attitude and acceptance of and participation in influenza vaccination by HCPs. In Saudi Arabia, the influenza vaccine is not mandatory; however, the Scientific Committee for Influenza and Pneumococcal Vaccination (SCIPV) guidelines, which were established by the Saudi Thoracic Society (STS), recommend that both the public and HCPs receive the vaccine every year, particularly during influenza season, because the composition of the vaccine is modified annually. Both the Saudi Ministry of Health (MOH) and STS recommend that all HCPs should receive the influenza vaccine annually [9, 28]. This study is likely to provide current and up-to-date insights that may prove useful in assessing the impact of the current interventions used to improve influenza vaccination coverage in Saudi Arabia. Several studies have reported hesitancy, inadequate knowledge and poor uptake of influenza vaccine among HCPs in several parts of the world, including Saudi Arabia [29–31]. A previous study conducted in Saudi Arabia found that the vaccination rate was 38% [31]. However, our literature search showed no current data for the 2016 winter season in Saudi Arabia. Thus, we conducted a study to determine the attitudes, knowledge, acceptance of and participation in HCP influenza vaccination in the north and central regions of Saudi Arabia. Additionally, to assess the effect of the influenza vaccine campaign, following the recommendations of our previous study, we assessed the vaccination rate among HCPs in Saudi Arabia during the 2012–2013 influenza season [31]. This study will be helpful for better planning and policies regarding influenza vaccination in Saudi Arabia.

## Methods

### Study tool and design

A cross-sectional study was conducted in 8 major government-run and private hospitals and their primary care centers at distinct locations in the cities of Riyadh and Hail in Saudi Arabia in October–November 2016. A validated questionnaire composed of 31 close-ended questions and 5 sections was used. The first section discussed questions related to the demographics of participants. In the second section, there were questions about their attitude toward vaccination, such as if they routinely get vaccinated and the reason why they do not get vaccinated. Based on their answers, the participants were stratified into two groups (i.e., vaccinated and unvaccinated). Their knowledge was assessed in the third section through several questions about vaccine effectiveness and current guidelines related to vaccination in Saudi Arabia as well as internationally. The fourth section discussed questions related to HCP practices, such as the current practice in their hospitals with respect to vaccine administration and offering the vaccine to HCPs and patients. In the final section, questions about vaccine awareness, such as accessibility to the vaccine, current guidelines, associated risk and symptoms of infection, were discussed (see Additional file 1).

For validation, the questionnaire was assessed by a pilot study of 10 experienced HCPs with both a clinical and research background to determine if the questions were valid, clear and understandable. The questionnaire was partially modified on the basis of the results obtained, and these modifications were in the English language. The data collected in pilot study was excluded from the final results. The questionnaire was adopted from a published study and is in English language since the study population is HCPs.

### Data collection

A total of 405 self-administered questionnaires were distributed among the HCPs of hospitals, and the HCPs were recruited using a simple sampling technique. The questionnaire was anonymous. The data collection was completed during October–November, 2016 by four trained Pharm D senior students. The data collectors obtained the complete data in 3 visits. The data collector explained the study and its purpose to each HCP and informed them that participation was voluntary. Accepting and returning a completed questionnaire was taken as consent to participate in the study. During the first visit to the study sites, the questionnaires were distributed to all sampled HCPs who were invited and consented to participate, and the completed questionnaires were collected within 5 days. The data collectors made a second visit to the study site within three days to ensure maximum participation of the sampled HCPs. In addition, the data collectors made a third visit and conducted face-to-face interviews to complete the questionnaires for some HCPs who consented to participate and received the questionnaires but, due to their busy schedule, could not complete the questionnaires.

### Power calculation

To calculate the sample size of the study, a 95% confidence level was used, and the absolute error was estimated to be 5%. In addition, a previous study was conducted under similar settings, which found that the influenza vaccination rate of HCPs was approximately 38%. All previous information was used to calculate the sample size for this study, which yielded a required sample size of 364 participants [31–33].

### Statistical analysis

The data was entered and analyzed using the Statistical Analysis Software (SAS®) version 9.2. The frequencies of survey items were described by descriptive statistical analysis. The categorical variables were compared by the chi-square test. The dependent variable was the vaccination status (uptake) and it was dichotomous (i.e., vaccinated or unvaccinated). The relationship between the dependent variable (vaccination status) and the independent variables was determined by bivariate analysis such as the chi-square test or independent t-test. Those independent variables that were significantly associated with the dependent variable were entered into the logistic regression to determine the predictors for vaccination status or uptake. The level of statistical significance was set at *P* < 0.05.

## Results

A total of 364 HCPs responded completely to the study, with a response and completion rate of 90%. A large proportion (61.8%) of participants were female. Regarding profession, the majority (60.4%) of the participants were nurses followed by physicians and pharmacists (14.8 and 12.6%, respectively) as shown in Table 1. Furthermore, most of the participating HCPs (33.8%) were general practitioners. While there was comparable participation between government-run and private institutions, there were slightly more government-run institutions (57.7% vs 43.3%).

**Table 1:** Demographics of healthcare professionals

Characteristic	Category	Frequency (%)
Gender	Male	139 (38.2)
	Female	225 (61.8)
Profession	Physician	54 (14.8)
	Pharmacist	46 (12.6)
	Nurse	220 (60.4)
	Laboratory scientist	15 (4.1)
	Others	28 (7.9)
Specialty	General practice	123 (33.8)
	Pediatrics	21 (5.8)
	Family medicine	7 (1.9)
	Internal medicine	58 (15.9)
	Obstetrics & gynecology	11 (3.0)
	Geriatrics	2 (0.5)
	Pharmacy	46 (12.6)
	Other	96 (26.4)
Place of practice	Government-run hospital	210 (57.7)
	Private hospital	154 (42.3)

Among respondents, 67.6% of HCPs were vaccinated. Of those, there were no significant differences in the vaccination rate among the various professions (physicians 68%, pharmacists 72% and nurses 69%, *P* = 0.29). With respect to each profession, there was a significant difference between the vaccinated HCPs and unvaccinated HCPs (e.g., vaccinated physicians 68% vs unvaccinated physician 32%, *P* < 0.0001).

**Fig. 1:**
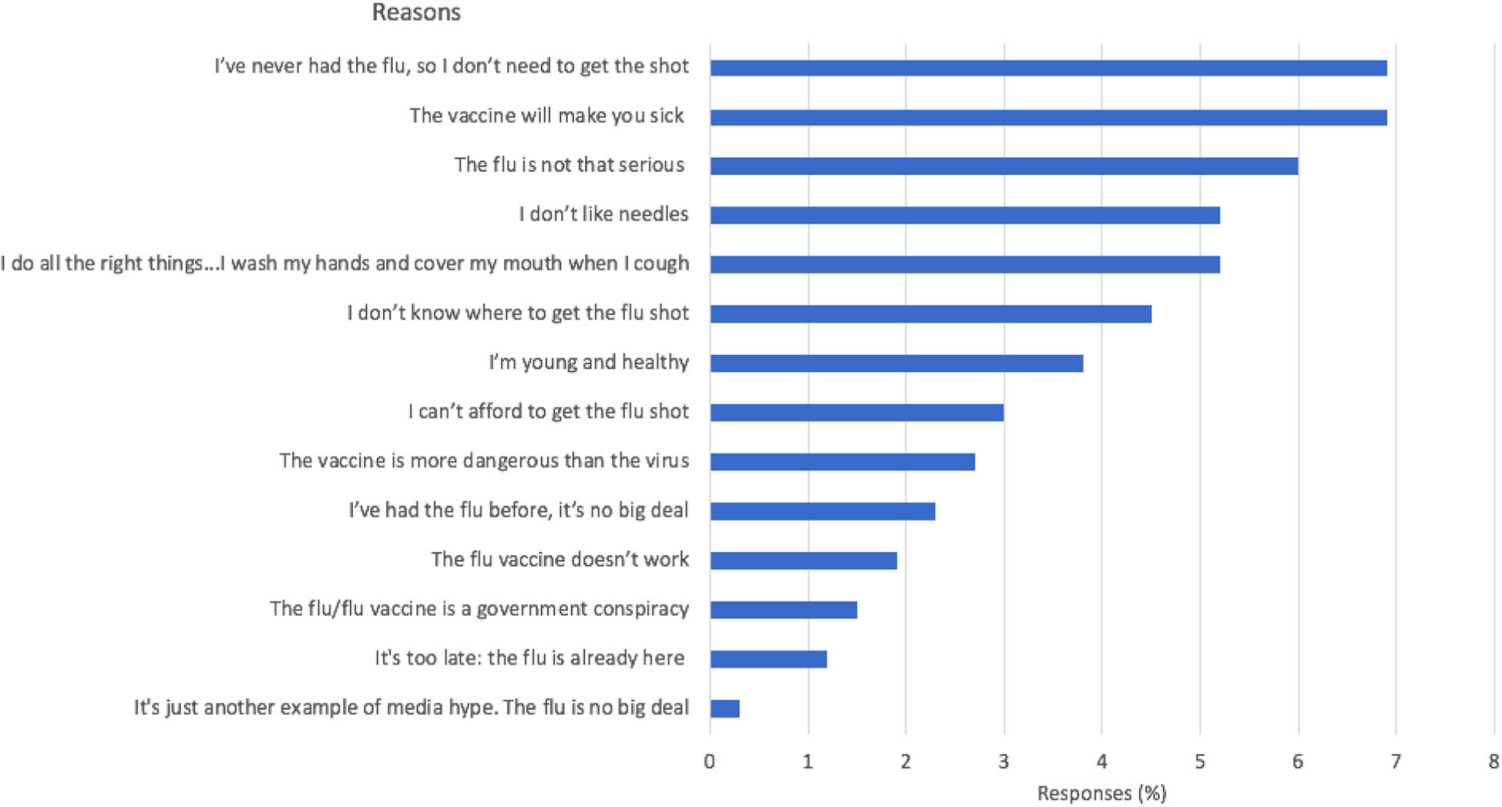
Reasons for rejecting routine vaccination

Approximately 7% of participants did not get vaccinated because of fear of sickness, and 7% did not routinely get vaccinated because they believed that they have never had influenza, followed by approximately 5.2% of participants who did not get vaccinated because they follow precautionary procedures (i.e., washing hands and covering their nose and mouth during sneezing) Fig. 1.

The majority (84.1%) of participants believed that the influenza vaccine is effective in preventing influenza infection, and of these, 94% were physicians, followed by pharmacists (89%) and nurses (83%) (*P* = 0.01). In terms of vaccination uptake, 74% of the HCPs who believed that the vaccine is effective for preventing influenza infection were vaccinated.

Approximately 58.7% of HCPs believed that the Saudi SCIPV guidelines recommend that HCPs should receive the flu shot. Of these HCPs, physicians comprised the highest percentage (65%), followed by nurses (61%) and pharmacists (50%). Nearly 62% of HCPs think the flu vaccine should be administered annually as shown in Table 2.

**Table 2:** Knowledge of healthcare professionals

Question	Response	Frequency (%)	*P value
Do you think the influenza vaccine is effective in preventing the flu?	Yes	306 (84.1)	< 0.001
	No	58 (15.9)	
Do you believe that the Saudi Scientific Committee for Influenza and Pneumococcal Vaccination (SCIPV) recommends that health care workers receive the flu shot?	Yes	213 (58.7)	< 0.001
	No	29 (8.0)	
	I do not know	121 (33.3)	
How often do you think the flu vaccine should be administered?	Every 6 months	83 (22.8)	< 0.001
	Every year	225 (61.8)	
	Every 5 years	19 (5.2)	
	Once in a lifetime	37 (10.2)	

*Chi square test

The majority of HCPs (80.2%) believed that the influenza vaccine should be part of the medical practice for HCPs (*P* < 0.001). Furthermore, 90% of physicians believed that the influenza vaccine should be part of medical practice, followed by nurses (81%) and pharmacists (76%), compared to those who did not believe from the same profession. In addition, 72% of the vaccinated HCPs believed that the influenza vaccine should be part of medical practice versus 27% of unvaccinated HCPs, and approximately 42.0% of HCPs believed that flu vaccine reduces the duration of disease. Only 34.9% of the HCPs’ medical centers require and offer the influenza vaccine, and approximately 16% do not offer the vaccine. Thirty-five percent of HCPs consider it a protective measure for patients, and 13.5% deem it an exemplary practice for other workers.

Approximately 70% of the HCPs did not attend any training related to the influenza vaccine; however, 65.1% of the HCPs are interested in attending these trainings. A large number of the centers (63.7%) are offering the influenza vaccine to the patients. Nevertheless, only half of the centers have standing orders regarding the influenza vaccine (Table 3).

**Table 3:** Practice patterns regarding influenza vaccination

Question	Response	Frequency (%)	**P value
Do you think administering the influenza vaccine should be part of your medical practice?	Yes	292 (80.2)	< 0.001
	No	70 (19.2)*	
Which statement applies to your practice/center regarding influenza vaccine for office staff?	We require and offer the influenza vaccine	127 (34.9)	0.03
	We encourage and offer the influenza vaccine	157 (43.1)	
	We require, but do not offer, the influenza vaccine	14 (3.8)	
	We encourage, but do not offer, the influenza vaccine	44 (12.1)	
	None of the above	22 (6.0)	
Have you or your staff participated in any training or continuing education related to the influenza vaccine in the past 12 months?	Yes	107 (29.4)	< 0.001
	No	257 (70.6)	
Would you or your staff be interested in participating in training related to the influenza vaccine?	Yes	237 (65.1)	< 0.001
	No	127 (34.9)	
Does your practice/center offer the influenza vaccine to your patients?	Yes	232 (63.7)	< 0.001
	No	39 (10.7)	
	I do not know	93 (25.5)	
Does your practice/center have standing orders regarding the influenza vaccine?	Yes	186 (51.1)	< 0.001
	No	59 (16.2)	
	I do not know	119 (32.7)	

* 3 participants did not respond to this question

** Chi square test

As for communication to the patients with respect to promotion of the influenza vaccine, approximately 50% of HCPs promote influenza vaccination through a poster or brochure, 15.4% by text reminder, 7.1% by telephone calls, and 5.2% by email reminder (*P* < 0.001). Furthermore, approximately 36.8% of HCPs promote it during patient visits, and 31.3% of HCPs promote a designated vaccination day (*P* ≤ 0.001). Less than 7% use some other source to promote vaccination. Unfortunately, 8.8% of HCPs are not engaged in promotional activities for the influenza vaccine. Among those who were vaccinated, 55% promoted vaccination through a poster or brochure, followed by 37% during patient visits, 33% during vaccination day and 20% through text reminders.

**Fig. 2:**
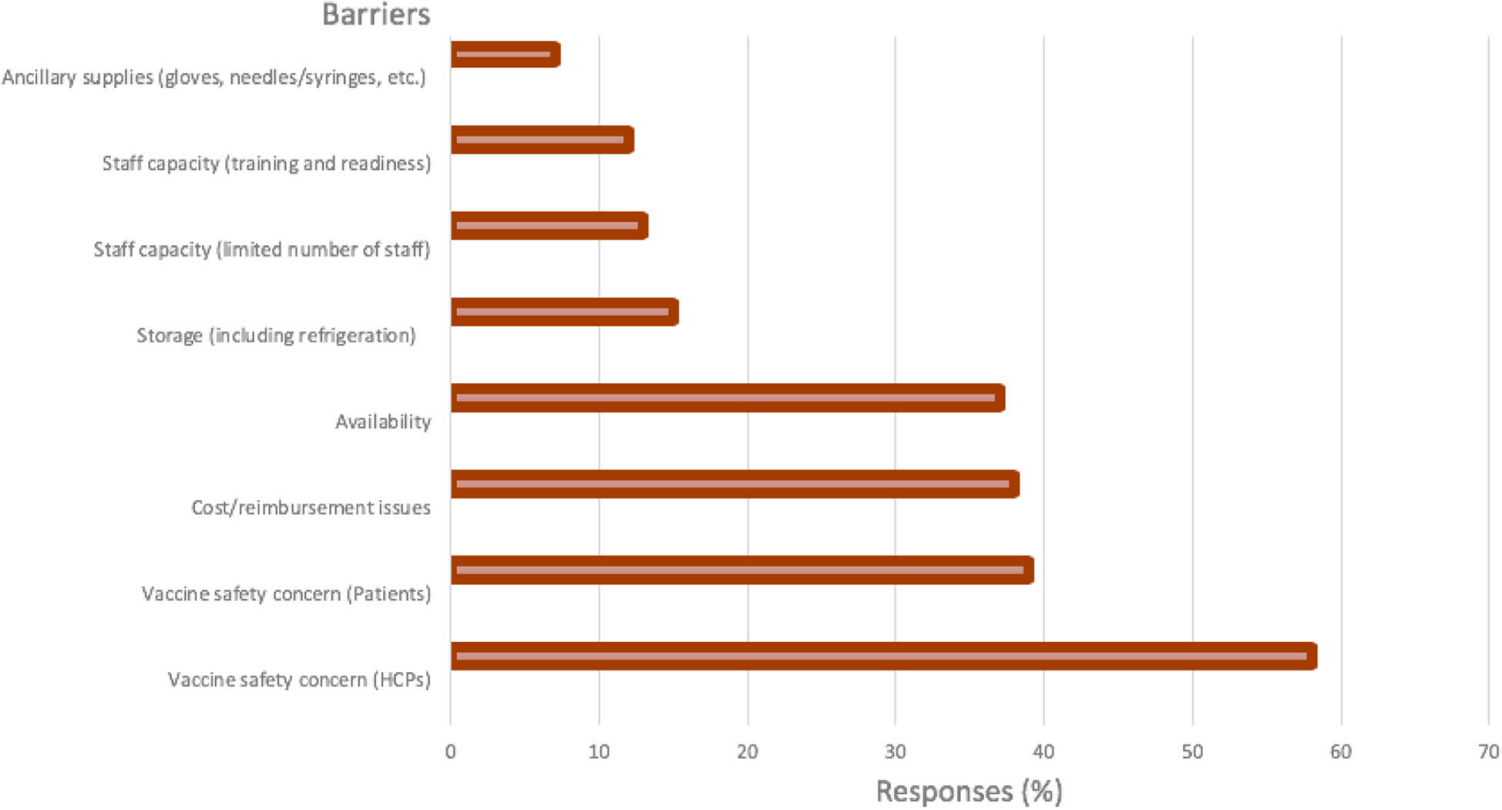
Barriers with regard to the vaccination rate

Approximatly 37% of HCPs believe that availability of the vaccine is a big barrier in the vaccination rate, and also cost/reimbursement issues (38%). Furthermore, vaccine safety concerns of HCPs (58.0%) was the highest contributing barrier of vaccination, as given in Fig. 2.

Table 4 shows that 74.2% of the HCPs believed that influenza is more serious than a “common cold”, and 48% of the HCPs do believe that patients can transmit influenza even if they are asymptomatic. Interestingly, approximately 20% of the HCPs who participated in this study thought that HCPs are less susceptible to influenza infection than others.

**Table 4:** Level of awareness regarding disease and vaccination

Question	Response frequency (%)			*P value with respect to categories
	Correct	Incorrect	Not sure	
HCPs are less susceptible to influenza infections than other people	69 (19.0)	275 (75.5)	20 (5.5)	< 0.001
Influenza is transmitted primarily by coughing and sneezing	318 (87.4)	19 (5.2)	27 (7.4)	< 0.001
Influenza is more serious than a “common cold”	270 (74.2)	48 (13.2)	46 (12.6)	< 0.001
The signs and symptoms of influenza include fever, headache, sore throat, cough, nasal congestion, and aches and pains	326 (89.6)	5 (1.4)	33 (9.1)	< 0.001
HCPs can spread influenza even when they are feeling well	220 (60.4)	52 (14.3)	92 (25.3)	< 0.001
People with influenza can transmit the infection only after their symptoms appear	125 (34.3)	176 (48.4)	63 (17.3)	< 0.001
Influenza is transmitted primarily by contact with blood and body fluids	106 (29.1)	187 (51.4)	106 (19.5)	< 0.001
Influenza vaccination may not work if the vaccine contains the wrong mix of viruses	244 (67.0)	26 (7.1)	94 (25.8)	< 0.001
The flu shot contains live viruses that may cause some people to get influenza	154 (42.3)	125 (34.3)	85 (23.4)	< 0.001
Influenza vaccination does not work in some persons, even if the vaccine has the right mix of viruses	201 (55.2)	52 (14.3)	111 (30.5)	< 0.001
Adults with influenza commonly experience nausea and vomiting or diarrhea	184 (50.5)	89 (24.5)	91 (25.0)	< 0.001
Symptoms typically appear 8 to 10 days after a person is exposed to influenza	125 (34.3)	105 (28.8)	134 (36.8)	0.163

* Chi square test

Table 5 shows the results of the multivariate logistic regression analyses. HCPs’ belief that vaccination prevent influenza infection (odds ratio (OR) = 3.93, 95% confidence interval (CI) = 1.97–7.82), their awareness of the guidelines and recommendations from the current advisory committee on immunization practices and the SCIPV (OR = 2. 13, 95% CI = 1.16–3.90) and the presence of standing orders regarding the influenza vaccine (OR = 1.57, 95% CI = 1.01–3.21), were the predictors for HCPs’ acceptance of the influenza vaccine.

**Table 5:** Logistic regression analysis identifying predictors for receiving the influenza vaccine among the participating healthcare professionals

	Odds Ratio (OR)	95% confidence interval (CI)
Gender		
Female (reference)	-	-
Male	1.404	0.784–2.514
Profession		
(Physician, reference)	-	-
Pharmacist	1.075	0.414–2.790
Nurse	1.461	0.659–3.241
Practice site		
(Private hospital, reference)	-	-
Governmental hospital	1.170	0.677–2.023
Vaccine is effective in preventing influenza		
No (reference)	-	-
Yes	3.934	1.979–7.820
Influenza vaccine should be part of your medical practice		
No (reference)	-	-
Yes	1.326	0.684–2.574
Have standing orders regarding the influenza vaccine		
No (reference)		
Yes	1.570	1.017–3.214
I don’t know	0.927	0.455–1.886
Aware of ACIP, SCIPV or CDC recommendations		
No (reference)	-	-
Yes	2.131	1.163–3.907

## Discussion

This study investigated the knowledge, attitude, and practice of HCPs working in Saudi hospitals and primary care centers regarding influenza vaccines during the 2016 season. This study had a high response rate (90%), which is similar to other studies performed in the same region [31, 34].

The current study shows an increase in influenza vaccination of HCPs, at 67.6%, in the 2016 season in the central part of Saudi Arabia. This vaccination rate is considerably higher than previous years. Indeed, the current vaccinate rate (67.6%) represents an approximate 43% increase over the vaccination rate reported among HCPs (38%) in a previous study conducted in similar settings in the 2013 season [31]. The increase in the vaccination rate in this study compared with that in a previous study done in Saudi Arabia could be due to the adoption of recommendations from the previous study by the ministry of health. Furthermore, our finding is comparable to a Kuwaiti study that reported a similar rate of 67.2% [35] and appears consistent with trends in a developed setting [36–38]. This study also reveals a higher vaccination rate among HCPs than other similar studies conducted in African (6.5%) and Asian (17.0%) countries [39, 40].

The high level of acceptance and uptake of influenza vaccination reported by HCPs in Saudi Arabia is an exciting development with a potential positive impact on public health. It is well established that HCPs’ attitude and acceptance of and participation in influenza vaccination are consistently correlated to vaccination by patients and the level of public trust in the utilitarian value of vaccination in enhancing public health [18, 20, 41].

Furthermore, the increase in favorable attitudes of HCPs to routine influenza vaccination suggests highly motivated professionals who appear poised to enhance patients’ uptake of influenza vaccination with adequate education that will correctly guide informed vaccination-related decisions. This position is consistent with our finding that showed that a majority of the HCPs actively promote vaccination and educate patients about its associated health benefits during face-to-face interactions and via a variety of media, such as posters, brochures, short reminder messages service via mobile phones and telephone calls. These remarkable vaccine-friendly practices by this sample of HCPs can only strengthen the culture of vaccination and adoption of influenza vaccine specifically among Saudi patients and the public [11].

The factors underlining our finding that showed the remarkable increase in the HCP use of the influenza vaccine appear multifactorial. We identified regular availability and ease of access to influenza vaccine in about two-thirds of the study sites, and this appeared to be an important contributory factor. Ease of access to influenza vaccines is a potent factor that can enhance acceptance and use especially among HCPs with an intrinsic positive attitude to influenza vaccination and vice-versa. Indeed, limited access to the influenza vaccine was cited by approximately 37% of HCPs as a potential threat to high immunization coverage, and this is consistent with findings of other studies in India and Turkey as well as those in similar settings that have identified difficult access as a key factor underlining low influenza vaccination coverage among HCPs [29, 31, 40, 42–44].

Furthermore, a recent study in Saudi Arabia showed that other contributory factors may also be at play. These include institutional initiatives to more deeply engage HCPs, especially nurses, to champion the uptake of influenza vaccination by HCPs [45]. This is because studies have consistently shown that the influenza vaccination rate is higher among nurses relative to other HCPs in Saudi Arabia, and this trend is also consistent with our findings [45, 46].

In contrast to a study from Switzerland, this study indicates that nurses are well-positioned to champion influenza vaccination by other HCPs [10]. Hence, nurses appeared well-positioned to champion increased influenza vaccination by other HCPs. Perhaps, the larger proportion of nurses, relative to other HCPs, and their more frequent contact with patients increases their risk of exposure to infectious disease such as influenza. Indeed, concerns with perceived increased risk of exposure to infection have been reported as a key motivating factor for high influenza vaccine use among HCPs [29, 47, 48].

Hence, adopting nurses as “influenza vaccine champions” may be a simple but potentially effective strategy that could boost acceptance and use of the influenza vaccine among HCPs in similar healthcare settings. Furthermore, the progressive shift of organizational policy in some of the health institutions in Saudi Arabia from voluntary to mandatory requirement of the yearly uptake of influenza vaccination for HCPs is also probably contributory. However, this phenomenon was not specifically explored in our study, but it has been established that the institutionalized policy of mandatory rather than voluntary vaccination almost always increased the influenza vaccination rate among HCPs [45, 49, 50].

Despite the apparent incremental progress in the influenza vaccination rate of HCPs in Saudi Arabia, a considerable amount of work is required to sustain this progress. This is because our study identified some factors that, if not resolved, may increase the risk of vaccine hesitancy and threaten immunization coverage. Although, this was identified in a small proportion of HCPs, especially physicians, it nonetheless deserves serious attention. Similar to a French study, this study also indicates some of the barriers to the vaccination of HCPs [51]. These factors include absence of past influenza infection, concerns that influenza vaccination may lead to sickness, false sense of protection from influenza infection due to strict adherence to hand-washing and safety concerns with influenza vaccine. These findings are consistent with similar reports from several published studies and can potentially increase vaccine hesitancy among HCPs if left unresolved [30, 31, 38, 52]. Therefore, there remains a need for a functional institutional framework that regularly monitors HCPs for vaccine-unfriendly attitudes and beliefs and resolves any potential problems that could compromise the integrity of the annual influenza vaccination program.

Our study shows a general positive attitude toward influenza vaccination by the sampled HCPs, as a majority is confident in the efficacy of the influenza vaccine to protect against and reduce the duration of infection and recommends its uptake regularly to patients and other HCPs. This finding is similar to the trends observed in published studies among HCPs with good acceptance and uptake of influenza vaccinations [29, 30, 48].

Furthermore, our study showed that approximately 70% of HCPs did not attend any specific training related to influenza infection and vaccine in recent years, though they express desire to do so. Published literature in the study area has not truly established a consistent correlation between HCP awareness of relevant updates related to vaccine and vaccination and the actual acceptance and uptake of influenza vaccination because of the complex and multilayered nature of behavioral and organizational factors associated with vaccine hesitancy [15, 17, 48]. It is nonetheless important to ensure that opportunities are provided to HCPs to regularly update their knowledge of current guidelines and other relevant information related to influenza infection and vaccine.

This study has several strengths and limitations. It was conducted in 8 major government-run and private hospitals and their primary clinics at distinct locations in the cities of Riyadh and Hail, covering both sectors and a relatively large number of hospitals. In addition, face-to-face interviews were conducted by the choice of HCPs and resulted in the participation of professionals from diverse professions. Therefore, this study illustrates vaccination coverage in different professions.

The low representation of physicians and pharmacists is one of the limitations to this study. The use of self-reporting for data gathering may be a possible limitation in the interpretation of the findings presented due to recall bias. However, published studies have established strong concordance between self-reported influenza vaccination status and the documented vaccination status in medical records, and hence this concern is unlikely and may be mitigated [53–55]. Furthermore, the high response and completion rate by the sampled HCPs, which ranks among the highest in recent times in Saudi Arabia, suggests that the findings presented probably provide a more valid snapshot of the HCPs’ attitude to and uptake of influenza vaccination in Saudi Arabia. As with most such studies (i.e., surveys), volunteer bias is one limitation that could affect the results of the study.

### Implications of the study findings

This study showed good vaccination coverage and improved HCP beliefs towards the influenza vaccine and vaccination. The study results might be a useful addition to the framework used to assess the impact of the efforts by the Saudi MOH, especially periodic campaigns, to improve vaccinations in Saudi Arabia. Furthermore, health institutions such as hospitals and centers should make vaccination of HCPs an institutionalized standard medical practice, ensure regular availability of the influenza vaccine and provide relevant training and education to HCPs to ensure a sustained increase in influenza vaccination coverage.

## Conclusion

The acceptance of and participation in influenza vaccination by HCPs in Saudi Arabia appears to have markedly increased in the 2016 season. However, there is still a need for appropriate interventions to sustain and subsequently enhance HCP participation rates and improve their knowledge, especially concerning important information about influenza infection and vaccination. This can be accomplished through continuing education and training programs for all categories of HCPs.

## Additional file

Additional file 1: Healthcare professionals’ knowledge, attitude and acceptance of influenza vaccination in Saudi Arabia Study Questionnaire. (PDF 127 kb)

## Abbreviations

CI: Confidence Interval
HCPs: Healthcare Professionals
MOH: Ministry of Health
OR: Odds Ratio
SAS: Statistical Analysis Software
SCIPV: Scientific Committee for Influenza and Pneumococcal Vaccination
STS: Saudi Thoracic Society

## References

[1] World Health Organization (WHO). Influenza Update, Number 321, 2018, July. Available:http://www.who.int/influenza/surveillance_monitoring/updates/EN_GIP_Influenza_transmission_zones.pdf. Accessed 20 August 2018.

[2] McLennan S, Wicker S (2010). Reflections on the influenza vaccination of healthcare workers. Vaccine..

[3] Iuliano AD, Roguski KM, Chang HH, Muscatello DJ, Palekar R, Tempia S (2018). Estimates of global seasonal influenza-associated respiratory mortality: a modelling study. Lancet..

[4] World Health Organization (WHO). Seasonal Influenza Fact Sheet. 2018, January. Available:https://www.who.int/news-room/fact-sheets/detail/influenza-(seasonal). Accessed 20 Aug 2018.

[5] Blank PR, Szucs TD (2009). Increasing influenza vaccination coverage in recommended population groups in Europe. Expert Rev Vaccines.

[6] Fiore AE (2009). Prevention and control of seasonal influenza with vaccines: recommendations of the advisory committee on immunization practice (ACIP). MMWR Recomm Rep.

[7] Pearson ML, Bridges CB, Harper SA (2006). Influenza vaccination of healthcare personnel: recommendation of the healthcare infection control practices advisory committee (HIPAC) and the advisory committee on immunization practices (ACIP). MMWR Recomm Rep.

[8] Centre for Disease Control and Prevention (CDC). Prevention strategies for influenza in healthcare settings. Guidelines and recommendations. Available: http://www.cdc.gov/flu/professionals/infectioncontrol/healthcaresettings.htm. Accessed 20 August 2018.

[9] Zeitouni MO, Al Barrak AM, Al-Moamary MS, Alharbi NS, Idrees MM, Al Shimemeri AA (2015). The Saudi thoracic society guidelines for influenza vaccinations. Ann Thorac Med.

[10] Pless A, McLennan SR, Nicca D, Shaw DM, Elger BS. Reasons why nurses decline influenza vaccination: a qualitative study. BMC Nurs 2017;16:20. 10.1186/s12912-017-0215-5 PMC5410084 28465672

[11] Opel DJ, Heritage J, Taylor JA, Mangione-Smith R, Salas HS, Devere V (2013). The architecture of provider-parent vaccine discussions at health supervision visits. Pediatrics..

[12] Jiménez-García R, Hernández-Barrera V, Carrasco-Garrido P, Sierra-Moros MJ, Martinez-Hernandez D (2006). Influenza vaccination coverages among Spanish children, adults and health care workers. Infection..

[13] Vaux S, Van Cauteren D, Guthmann JP, Le Strat Y, Vaillant V, de Valk H (2011). Influenza vaccination coverage against seasonal and pandemic influenza and their determinants in France: a cross-sectional survey. BMC Public Health.

[14] Kraut A, Graff L, McLean D (2011). Behavioral change with influenza vaccination: factors influencing increased uptake of the pandemic H1N1 versus seasonal influenza vaccine in health care personnel. Vaccine..

[15] MacDonald NE. SAGE Working Group on Vaccine Hesitancy. Vaccine hesitancy: definition, scope and determinants. Vaccine. 2015.04.036. 10.1016/j.vaccine. 10.1016/j.vaccine.2015.04.036 25896383

[16] Wheeler M, Buttenheim AM (2013). Parental vaccine concerns, information source, and choice of alternative immunization schedules. Hum Vaccin Immunother..

[17] Verger P, Fressard L, Collange F, Gautier A, Jestin C, Launay O (2015). Vaccine hesitancy among general practitioners and its determinants during controversies: a National Cross-sectional Survey in France. EBioMedicine..

[18] Schwarzinger M, Verger P, Guerville MA, Aubry C, Rolland S, Obadia Y (2010). Positive attitudes of French general practitioners towards a/H1N1 influenza-pandemic vaccination: a missed opportunity to increase vaccination uptakes in the general public?. Vaccine..

[19] Asma S, Akan H, Uysal Y, Pocan AG, Sucakli MH, Yengil E (2016). Factors effecting influenza vaccination uptake among health care workers: a multi-center cross-sectional study. BMC Infect Dis.

[20] Dube E, Laberge C, Guay M, Bramadat P, Roy R, Bettinger J (2013). Vaccine hesitancy: an overview. Hum Vaccin Immunother..

[21] Bean SJ, Catania JA (2013). Vaccine perceptions among Oregon health care providers. Qual Health Res.

[22] MacDonald NE, Dube E (2015). Unpacking vaccine hesitancy among healthcare providers. EBioMedicine..

[23] Favin M, Steinglass R, Fields R, Banerjee K, Sawhney M (2012). Why children are not vaccinated: a review of the grey literature. Int Health.

[24] Pless A, Shaw D, McLennan S, Elger BS (2017). Nurses' attitudes towards enforced measures to increase influenza vaccination: a qualitative study. Influenza Other Respir Viruses.

[25] Gatrad AR, Sheikh A (2005). Hajj: journey of a lifetime. BMJ..

[26] Khan K, Memish ZA, Chabbra A, Liauw J, Hu W, Janes DA (2010). Global public health implications of a mass gathering in Mecca, Saudi Arabia during the midst of an influenza pandemic. J Travel Med.

[27] Al-Nozha MM, Abdullah M, Arafah MR, Khalil MZ, Khan NB, Al-Mazrou YY (2007). Hypertension in Saudi Arabia. Saudi Med J.

[28] Seasonal Influenza Vaccination. Ministry of Health. https://www.moh.gov.sa/en/Flu/Pages/QA.aspx. Accessed on Dec 17th, 2018.

[29] Hollmeyer HG, Hayden F, Poland G, Buchholz U (2009). Influenza vaccination of health care workers in hospitals--a review of studies on attitudes and predictors. Vaccine..

[30] Rehmani R, Memon JI (2010). Knowledge, attitudes and beliefs regarding influenza vaccination among healthcare workers in a Saudi hospital. Vaccine..

[31] Alshammari TM, AlFehaid LS, AlFraih JK, Aljadhey HS (2014). Health care professionals' awareness of, knowledge about and attitude to influenza vaccination. Vaccine..

[32] Charan J, Biswas T (2013). How to calculate sample size for different study designs in medical research?. Indian J Psychol Med.

[33] Pourhoseingholi MA, Vahedi M, Rahimzadeh M (2013). Sample size calculation in medical studies. Gastroenterol Hepatol Bed Bench.

[34] Haridi HK, Salman KA, Basaif EA, Al-Skaibi DK (2017). Influenza vaccine uptake, determinants, motivators, and barriers of the vaccine receipt among healthcare workers in a tertiary care hospital in Saudi Arabia. J Hosp Infect.

[35] Abu-Gharbieh E, Fahmy S, Rasool BA, Khan S (2010). Influenza vaccination: healthcare workers attitude in three Middle East countries. Int J Med Sci.

[36] Miller BL, Ahmed F, Lindley MC, Wortley PM (2011). Increases in vaccination coverage of healthcare personnel following institutional requirements for influenza vaccination: a national survey of U.S. hospitals. Vaccine..

[37] Picazo JJ (2012). González F, Salleras L, Bayas JM, Alvarez MJ (2012). Survey of adult influenza and pneumococcal vaccination in Spain. Vacunas..

[38] Kent JN, Lea CS, Fang X, Novick LF, Morgan J (2010). Seasonal influenza vaccination coverage among local health department personnel in North Carolina, 2007-2008. Am J Prev Med.

[39] James PB, Rehman IU, Bah AJ, Lahai M, Cole CP, Khan TM (2017). An assessment of healthcare professionals' knowledge about and attitude towards influenza vaccination in Freetown Sierra Leone: a cross-sectional study. BMC Public Health.

[40] Khan TM, Khan AU, Ali I, Wu DB (2016). Knowledge, attitude and awareness among healthcare professionals about influenza vaccination in Peshawar, Pakistan. Vaccine..

[41] Leask J, Willaby HW, Kaufman J (2014). The big picture in addressing vaccine hesitancy. Hum Vaccin Immunother.

[42] Charrel RN, Nougairede A, Brouqui P, Raoult D, Gautret P (2015). Influenza vaccine for hajj and Umrah pilgrims. Lancet Infect Dis.

[43] Bali NK, Ashraf M, Ahmad F, Khan UH, Widdowson MA, Lal RB (2013). Knowledge, attitude, and practices about the seasonal influenza vaccination among healthcare workers in Srinagar, India. Influenza Other Respir Viruses.

[44] Ozisik L, Tanriover MD, Altinel S, Unal S (2017). Vaccinating healthcare workers: level of implementation, barriers and proposal for evidence-based policies in Turkey. Hum Vaccin Immunother..

[45] Al-Otaibi BM, El-Saed A, Balkhy HH (2010). Influenza vaccination among healthcare workers at a tertiary care hospital in Saudi Arabia: facing challenges. Ann Thorac Med..

[46] Alabbad AA, Alsaad AK, Al Shaalan MA, Alola S, Albanyan EA (2018). Prevalence of influenza vaccine hesitancy at a tertiary care hospital in Riyadh, Saudi Arabia. J Infect Public Health.

[47] La Torre G, Mannocci A, Ursillo P, Bontempi C, Firenze A, Panico MG (2011). Prevalence of influenza vaccination among nurses and ancillary workers in Italy: systematic review and meta analysis. Hum Vaccin.

[48] Dominguez A, Godoy P, Castilla J, Soldevila N, Toledo D, Astray J (2013). Knowledge of and attitudes to influenza vaccination in healthy primary healthcare workers in Spain, 2011-2012. PLoS One.

[49] Christini AB, Shutt KA, Byers KE (2007). Influenza vaccination rates and motivators among healthcare worker groups. Infect Control Hosp Epidemiol.

[50] Steckel CM (2007). Mandatory influenza immunization for health care workers--an ethical discussion. AAOHN J.

[51] Hulo S, Nuvoli A, Sobaszek A, Salembier-Trichard A (2017). Knowledge and attitudes towards influenza vaccination of health care workers in emergency services. Vaccine..

[52] Sundaram N, Duckett K, Yung CF, Thoon KC, Sidharta S, Venkatachalam I (2018). "I wouldn't really believe statistics" - challenges with influenza vaccine acceptance among healthcare workers in Singapore. Vaccine..

[53] Bouadma L, Barbier F, Biard L, Esposito-Farese M, Le Corre B, Macrez A (2012). Personal decision-making criteria related to seasonal and pandemic a(H1N1) influenza-vaccination acceptance among French healthcare workers. PLoS One.

[54] Zimmerman RK, Raymund M, Janosky JE, Nowalk MP, Fine MJ (2003). Sensitivity and specificity of patient self-report of influenza and pneumococcal polysaccharide vaccinations among elderly outpatients in diverse patient care strata. Vaccine..

[55] Loulergue P, Moulin F, Vidal-Trecan G, Absi Z, Demontpion C, Menager C (2009). Knowledge, attitudes and vaccination coverage of healthcare workers regarding occupational vaccinations. Vaccine..

