# Supplementary material for: Healthcare professionals’ knowledge, attitude and acceptance of influenza vaccination in Saudi Arabia: a multicenter cross-sectional study
Source: BMC Health Serv Res. 2019 Apr 15;19:229. doi: 10.1186/s12913-019-4054-9 (PMC6469139; doi:10.1186/s12913-019-4054-9)
Supplement: Supplementary file 1 — Healthcare professionals’ knowledge, attitude and acceptance of influenza vaccination in Saudi Arabia Study Questionnaire. (PDF 127 kb) [file 12913_2019_4054_MOESM1_ESM.pdf]

The Health Care Professional's knowledge, Attitude, and Acceptance toward Influenza Vaccines in Saudi Hospitals

Thank you for participation in this study. The aim of this questionnaire is to study the opinion and attitude toward seasonal influenza vaccination, which may help to find solutions aimed to improve influenza vaccination rates among heath care professionals in Saudi Arabia

Please be advised that all information will be dealt with in strict confidence and will be used for research purposes only.

Please mark the appropriate box next to your answer choice with an "x". Please answer all of the questions to the best of your ability

General Information

1. Gender:

☐ Male

☐ Female

2. What is your professional title?

☐ Physician

☐ Pharmacist

☐ Nurse

☐ Lab specialties

☐ Other (please specify)______________________

3. What is your specialty?

☐ General practice

☐ Pediatrics

☐ Family Medicine

☐ Internal Medicine

☐ OBGYN

☐ Geriatrics

☐ Asthma/Allergies

☐ Pharmacy

☐ Other (please specify)_______________________

4. What is your practice/center?

☐ Governmental hospital

☐ Private hospital

☐ Other (please specify)_______________________

Question measure attitude

5. Do you routinely get vaccinated against influenza? And if No please check question 6?

☐ Yes

☐ No

6. **Check which statements pertain to why you do not routinely get the influenza vaccine (check all that apply)**

- ☐ The vaccine will make you sick
- ☐ I've never had the flu, so I don't need to get the shot
- ☐ I do all the right things...I wash my hands and cover my mouth when I cough
- ☐ I've had the flu before, it's no big deal
- ☐ I don't like needles
- ☐ The flu/flu vaccine is a government conspiracy
- ☐ The flu is not that serious
- ☐ The vaccine is more dangerous than the virus
- ☐ I'm young and healthy
- ☐ I can't afford to get the flu shot
- ☐ I don't know where to get the flu shot
- ☐ The flu vaccine doesn't work
- ☐ It's just another example of media hype. The flu is no big deal
- ☐ It's too late: the flu is already here
- ☐ Other (please specify)

<u>Questions measure knowledge</u>

7. **Do you think the influenza vaccine is effective in preventing the flu?**

- ☐ Yes
- ☐ No

8. **Do you believe that Saudi Scientific Committee for Influenza and Pneumococcal Vaccination (SCIPV) recommends that health care workers receive the flu shot?**

- ☐ Yes
- ☐ No
- ☐ I don't know

9. **How often do you think the flu vaccine should be administered? Choose one**

- ☐ Every 6 months
- ☐ Every year
- ☐ Every 5 years
- ☐ Once in a lifetime

<u>Questions about Practice</u>

10. **Do you think administering the influenza vaccine should be part of your medical practice?**

- ☐ Yes
- ☐ No

11. **Which statement applies to your practice/center regarding influenza vaccine for office staff? (Check only one)**

- ☐ We require and offer the influenza vaccine
- ☐ We encourage and offer the influenza vaccine
- ☐ We require, but do not offer the influenza vaccine
- ☐ We encourage, but do not offer the influenza vaccine
- ☐ None of the above

12. **Why are flu vaccines for health care workers encouraged? (Choose all that apply)**

- ☐ To minimize sick days and loss of productivity
- ☐ Because healthcare workers can get exposed to the flu by sick patients
- ☐ Because sick patients are exposed to the flu by healthcare workers
- ☐ To set an example to other workers

13. Have you or your staff participated in any training or continuing education related to the influenza vaccine in the past 12 months?

☐ Yes ☐ No

14. Would you or your staff be interested in participating in training related to the influenza vaccine?

☐ Yes ☐ No

15. Does your practice/center offer the influenza vaccine to your patients?

☐ Yes ☐ No

☐ I don't know

16. Does your practice/center have standing orders regarding the influenza vaccine?

☐ Yes ☐ No

☐ I don't know

17. How do you communicate the importance of getting the influenza vaccine to your patients? (Check all that apply):

☐ During office visits

☐ Telephone calls

☐ Poster or Brochure

☐ Vaccine reminders by e-mail

☐ Vaccine reminders by text

☐ We do not communicate the importance of getting the influenza vaccine

☐ Vaccination day

☐ Other (please specify) ______________________

18. In your opinion, what are some of the barriers that prevent your practice from providing the influenza vaccine to some or all of your patients? (Check all that apply)

☐ Vaccine safety concern (yours)

☐ Vaccine safety concerns (patients)

☐ Cost/reimbursement issues

☐ Availability

☐ Storage (including refrigeration)

☐ Staff capacity (limited number of staff)

☐ Staff capacity (training and readiness)

☐ Ancillary supplies (gloves, needles/syringes, etc.)

<u>Question measure awareness</u>

19. **Are you aware of the published guidelines Advisory Committee on Immunization Practices (ACIP), Scientific Committee for Influenza and Pneumococcal Vaccination (SCIPV) or CDC for influenza immunization?**

☐ Yes ☐ No

☐ Other (please specify-) __________

Questions	Correct	Incorrect	Not sure
Health care professionals(HCPs) are less susceptible to influenza infections than other people			
Influenza is transmitted primarily by coughing and sneezing			
Influenza is more serious than a “common cold”			
The signs and symptoms of influenza include fever, headache, sore throat, cough, nasal congestion, and aches and pains			
HCPs can spread influenza even when they are feeling well			
People with influenza can transmit the infection only after their symptoms appear			
Influenza is transmitted primarily by contact with blood and body fluids			
Influenza vaccination may not work if the vaccine contains the wrong mix of viruses			
The flu shot contains live viruses that may cause some people to get influenza			
Influenza vaccination does not work in some persons, even if the vaccine has the right mix of viruses			
Adults with influenza commonly experience nausea and vomiting or diarrhea			
Symptoms typically appear 8 to 10 days after a person is exposed to influenza			

THANK YOU….
